# Trimeric Small Interfering RNAs and Their Cholesterol-Containing Conjugates Exhibit Improved Accumulation in Tumors, but Dramatically Reduced Silencing Activity

**DOI:** 10.3390/molecules25081877

**Published:** 2020-04-18

**Authors:** Ivan V. Chernikov, Daniil V. Gladkikh, Ulyana A. Karelina, Mariya I. Meschaninova, Alya G. Ven’yaminova, Valentin V. Vlassov, Elena L. Chernolovskaya

**Affiliations:** Institute of Chemical Biology and Fundamental Medicine SB RAS, Novosibirsk 630090, Russia; chernikovivanv@gmail.com (I.V.C.); medulla35@gmail.com (D.V.G.); uljana@ngs.ru (U.A.K.); mesch@niboch.nsc.ru (M.I.M.); ven@niboch.nsc.ru (A.G.V.); valentin.vlassov@niboch.nsc.ru (V.V.V.)

**Keywords:** siRNA, TsiRNA, 2’-O-methyl modifications, cholesterol conjugates, Dicer-substrate siRNA, *MDR1*, biodistribution

## Abstract

Cholesterol derivatives of nuclease-resistant, anti-*MDR1* small-interfering RNAs were designed to contain a 2’-OMe-modified 21-bp siRNA and a 63-bp TsiRNA in order to investigate their accumulation and silencing activity in vitro and in vivo. The results showed that increasing the length of the RNA duplex in such a conjugate increases its biological activity when delivered using a transfection agent. However, the efficiency of accumulation in human drug-resistant KB-8-5 cells during delivery in vitro in a carrier-free mode was reduced as well as efficiency of target gene silencing. TsiRNAs demonstrated a similar biodistribution in KB-8-5 xenograft tumor-bearing SCID mice with more efficient accumulation in organs and tumors than cholesterol-conjugated canonical siRNAs; however, this accumulation did not provide a silencing effect. The lack of correlation between the accumulation in the organ and the silencing activity of cholesterol conjugates of siRNAs of different lengths can be attributed to the fact that trimeric Ch-TsiRNA lags mainly in the intercellular space and does not penetrate sufficiently into the cytoplasm of the cell. Increased accumulation in the organs and in the tumor, by itself, shows that using siRNA with increased molecular weight is an effective approach to control biodistribution and delivery to the target organ.

## 1. Introduction

Small interfering RNAs (siRNA) are considered promising drugs that can effectively and selectively suppress the expression of genes associated with diseases. One of the unresolved problems of their use is the rapid elimination of such molecules from the bloodstream due to filtration by the kidneys, because their molecular weight lies below the filtration limit. Various approaches to increase their permanence in the bloodstream and improve accumulation in target organs are being actively developed on a worldwide basis. Such approaches include the formation of complexes with lipids and polymers [1,2], the attachment of ligands to siRNAs that interact with lipoproteins and other blood proteins [3], the use of specific ligands that facilitate the interaction of siRNAs with cells [4,5], as well as the development of formulations for their local administration and controlled release [6]. It is expected that the use of RNA duplexes with a higher molecular weight as inducers of RNA interference will improve the pharmacodynamics and pharmacokinetics of such drugs. It has previously been shown that introduction of chemical modifications (e.g., 2’-O-methyl) in the 2’ position of the ribose of G and U nucleotides significantly reduces the immunostimulatory activity of siRNAs in vitro and in vivo [7], which allows the use of RNA duplexes of almost any length, limited only by the technology and economy of synthesis, for therapeutic purposes. Previously, we found that 42- and 63-bp siRNAs containing 2′-OMe modifications in nuclease-sensitive sites induced more effective RNAi outcomes at lower concentrations than the classical 21-bp siRNAs without nonspecific immune effects acting in a Dicer-independent mode in cell culture after transfection [8]. Later, we designed a multimeric nuclease-resistant 63-bp trimeric small-interfering RNA (TsiRNA) comprising in one duplex the sequences of siRNAs targeting the mRNAs of the *MDR1*, *LMP2*, and *LMP7* genes [9]. The results showed that such TsiRNAs are able to suppress the expression of all their target genes independently and with high efficiency, acting via a Dicer-dependent mechanism. TsiRNA is diced into 42- and 21-bp duplexes inside the cell. TsiRNA-induced gene silencing is characterized by kinetics similar to that of canonical siRNAs, while the silencing efficiency is significantly higher than that of canonical siRNAs containing the same sequences.

Here, we obtained cholesterol derivatives of selectively 2’-OMe-modified 21-bp siRNA- and 63-bp TsiRNA targeted *MDR1* mRNA and investigated their accumulation and silencing activity in vitro and in vivo. We found that increasing the length of the RNA duplex in such conjugates increases their silencing activity when delivered using a transfection agent. However, there was a reduced efficiency of accumulation in cells and, accordingly, the observed suppression of the expression of the target gene during delivery in vitro in a carrier-free mode. In in vivo experiments with healthy and tumor-bearing mice, cholesterol-containing trimeric TsiRNAs demonstrated more efficient accumulation in organs and tumors than the same canonical siRNA derivatives; however, this accumulation did not provide an appreciable silencing effect.

## 2. Results and Discussion

Anti-*MDR1* monomeric and trimeric siRNAs and their conjugates with cholesterol connected though C6 linker (Table 1 and Table 2) were synthesized as described previously [9,10] The C6 linker was selected because the monomeric siRNA conjugate with this linker showed the highest biological activity compared to the conjugates with other linkers [10]. 2’-O-Methyl modifications were introduced into nuclease-sensitive sites according to the previously developed algorithm [11] in order to prevent degradation of carrier-free siRNA in the presence of serum and in the bloodstream. Monomeric anti-*MDR1* siRNA is homologous to the 557–577 nt region of human *MDR1* mRNA, demonstrated high silencing activity in our previous studies [10,12]. Two different trimeric siRNAs were used in this study: TsiRNA-1 containing the sequence of the monomeric siRNA repeated three times, which, as we showed earlier, possessed higher silencing activity than monomer when it was transfected into cells using Lipofectamine 2000. The second trimeric siRNA—TsiRNA-2 was composed of sequences of three siRNAs directed to different regions of the *MDR1* mRNA which was used for the accumulation assays using stem-loop PCR, since the presence of repeats with the composition of the first RNA hinders its accurate detection by this method. Previously, we showed that TsiRNA, in which all nuclease-sensitive sites were subjected to modifications, is highly resistant to ribonucleases, operates according to a Dicer-independent mechanism, and is not processed in a cell; TsiRNA containing fewer modifications can be processed by Dicer in a cell to 21 bp siRNAs, which act independently. To construct cholesterol derivatives, we chose the first variant with a large number of modifications in order to ensure the nuclease resistance of the conjugate in vivo.

### 2.1. In Vitro

First, the effect of the attachment of cholesterol to the 5′ end of the sense strand of trimeric siRNA on the its silencing activity was investigated under transfection conditions. The KB-8-5-MDR-GFP cell line, which was used to study the silencing activity [13], expresses a short-lived chimeric protein consisting of a fragment of P-glycoprotein (the product of the *MDR1* gene) and a GFP reporter protein, equipped with a rapid degradation signal. Both siRNAs and TsiRNA-1 effectively suppressed the expression of the target gene after transfection with Lipofectamine 2000, while the efficiency of the action of the trimeric TsiRNA was significantly higher than that of the monomeric siRNA (IC_50_ values were 3.8 nM for siRNA and 0.65 nM for TsiRNA-1 (Table 1)). Cholesterol conjugates of siRNA and TsiRNA showed an increase in IC_50_ values under transfection conditions by 8 and 25 times that of the corresponding non-conjugated siRNA and TsiRNA, respectively, suggesting that the attachment of cholesterol reduces their activity. The observed decrease in activity can be associated with both the influence of cholesterol attachment on the thermodynamics of the duplexes and on recognition of the conjugate by proteins of the RNA-interference machinery. A more significant decrease in the activity of TsiRNA-1 may be related to the fact that this trimer, as we have demonstrated previously [8], is not processed in the cell by Dicer due to the presence of 2′OMe modifications in the vicinity of expected sites of Dicer cleavage and TsiRNA-1 acts via a Dicer-independent mechanism, which may be more sensitive to modifications of the 5′ end of siRNA such as the attachment of cholesterol [9]. It was demonstrated [14] that 25–27 bp dsRNAs can be directly loaded into Ago2 and show better efficacy as compared with canonical 21-bp siRNAs; in this case, Ago2 protein may be more sensitive to the presence of chemical modifications, or it interacts with the 5′ end of the duplex sense strand. However, the cholesterol-conjugated TsiRNA-1 was still more active under transfection conditions than the siRNA conjugate with IC_50_ values of 16 and 29 nM, respectively.

The silencing activity of cholesterol-modified monomeric or trimeric siRNAs in a carrier-free mode was evaluated in two cell lines differing in the level of expression of the target gene. The drug-resistant line KB-8-5-MDR-GFP, which, along with the marker transgene *MDR1-GFP*, expresses at a high level endogenous *MDR1* and the drug-sensitive line KB-3-1-MDR-GFP, which expresses only the marker transgene and was obtained by lentiviral transduction and subsequent selection in a similar way.

**Figure 1:**
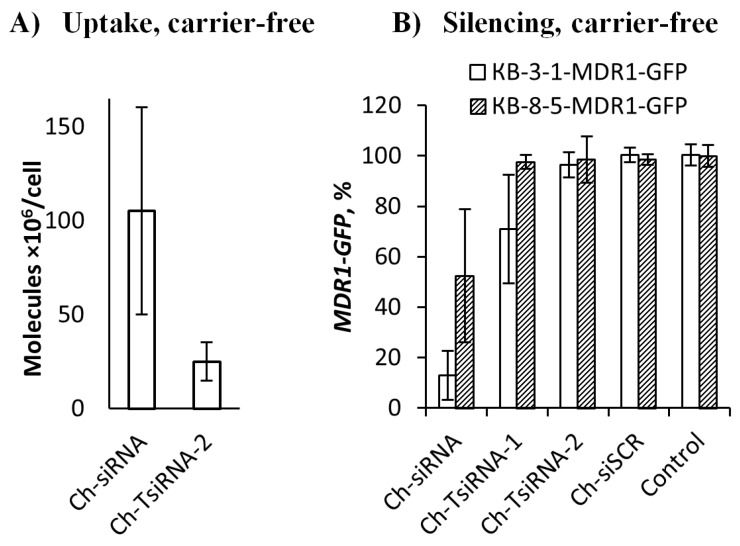
Accumulation (**A**) and silencing activity (**B**) of cholesterol-modified siRNAs and TsiRNAs in a carrier-free mode in vitro. (**A**) Accumulation of monomer (Ch-siRNA) and trimeric (Ch-TsiRNA-2) cholesterol-modified siRNAs in KB-8-5 cells 4 h after addition (1 µM), measured by stem-loop RT-PCR. B) Silencing activity of Ch-siRNA, Ch-TsiRNA-1 and Ch-TsiRNA-2 three days after addition (5 µM) to KB-8-5-MDR1-GFP or KB-3-1-MDR1-GFP cells, fluorescence intensity value of untreated cells was used as a 100%.

The study of the interaction of the conjugates with cells in a carrier-free mode revealed that Ch-siRNA accumulated 4.2 times more efficiently in cells than Ch-TsiRNA. The accumulation levels were 105 and 25 million molecules per cell, for Ch-siRNA and Ch-TsiRNA, respectively (Figure 1A). The observed reduction in the efficiency of accumulation in cells with an increase in the length of the conjugate may be related to its large size and decreased hydrophobicity. These factors can affect the interaction with the slightly negatively charged hydrophobic cell membrane, as well as cause steric difficulties during the internalization of the conjugate.

Determination of the silencing activity of the conjugates has shown that Ch-siRNA inhibits the expression of the target gene in KB-8-5-MDR1-GFP cells by at least 50%, while Ch-TsiRNA is practically inactive (Figure 1B). The efficiency of both conjugates in the KB-3-1-MDR1-GFP cell line was significantly higher. The monomeric Ch-siRNA reduced the expression of the target gene by 87%, whereas trimeric Ch-TsiRNA decreased the level of *MDR1-GFP* gene by 29% (Figure 1B). Such a difference in the observed silencing activity is most likely due to differences in the target mRNA copy number, since in KB-8-5-MDR1-GFP cells, the target for siRNA is both *MDR-GFP* mRNA and endogenous *MDR1* mRNA expressed at a high level. The data showed that although Ch-TsiRNA itself is more active than Ch-siRNA when delivered with a transfection agent (1.8 times difference in IC_50_), its ineffective penetration into cells in a carrier-free mode (4.2 times difference in accumulation) does not allow efficient suppression of target gene expression in carrier-free conditions in vitro. Nevertheless, the finding that the cholesterol conjugate of trimeric RNA is able to penetrate into a cell without a carrier and exert a silencing effect is a promising starting point for optimizing its transfection properties.

### 2.2. In Vivo

**Figure 2:**
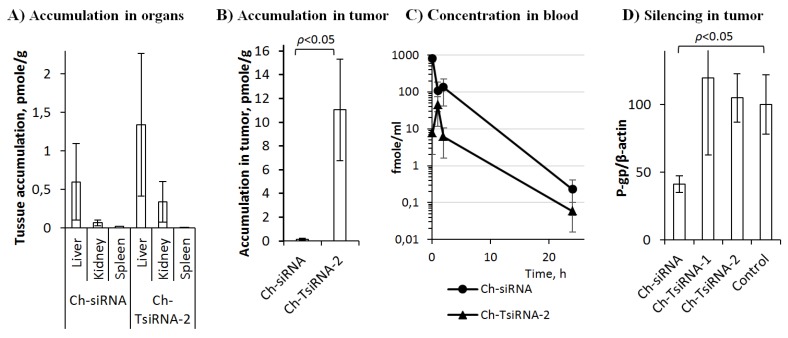
Biodistribution (**A**–**C**) and silencing activity in vivo (**D**) of cholesterol-modified siRNAs. (**A**) Biodistribution of Ch-siRNA and Ch-TsiRNA-2 in organs of mice 24 h after i.v. injection (2.5 nmole—1.7 and 5 µg/g, respectively). (**B**) Accumulation of Ch-siRNA and Ch-TsiRNA-2 in xenograft KB-8-5 tumors 24 h after i.v. injection (1.1 nmole—0.5 and 1.5 µg/g, respectively). (**C**) Concentration of Ch-siRNA and Ch-TsiRNA-2 in the blood of mice after i.v. injection of 2.5 nmole—1.7 and 5 µg/g, respectively. (**D**) Silencing activity of Ch-siRNA, Ch-TsiRNA-1 and Ch-TsiRNA-2 in xenograft KB-8-5 tumors in 6 days after i.v. injection (8.5 µg/g).

The effectiveness of the accumulation of therapeutic nucleic acids in culture and in vivo can differ significantly, since in the body they have to overcome additional barriers to the target organ and the effectiveness of this process can have a critical influence on the result of their action. We analyzed the biodistribution of Ch-siRNA and Ch-tsiRNA-2 in the organs of healthy SCID mice 24 h after intravenous (i.v.) administration at a dose of 2.5 nmol (corresponding to 1.7 and 5 µg/g for Ch-siRNA and Ch-TsiRNA, respectively) (Figure 2A).

Quantitative data were obtained by stem-loop PCR using calibration curves with known concentrations of the studied drugs added to samples of the corresponding organs of untreated mice. Ch-TsiRNA-2 accumulated in the liver and kidneys at least 2 times more efficiently (1.3 and 0.3 pmole/g, respectively) than Ch-siRNA (0.6 and 0.07 pmole/g, respectively), and the amounts of preparations accumulated in the spleen were similar and rather low (0.009 and 0.02 pmole/g). Ch-siRNA and Ch-TsiRNA-2 tumor accumulation analysis was performed on SCID mice with KB-8-5 xenograft tumors 24 h after i.v. administration in a dose of 1.1 nmole (0.5 and 1.5 µg/g, respectively). Ch-TsiRNA-2 accumulated much more effectively in the tumor, compared to Ch-siRNA, with differences of 75 times in weight or 25 times in terms of moles (Figure 2B). The accumulation of siRNA preparations in organs is mainly determined by the duration of their presence in the bloodstream and the characteristic of organ vasculature [15]. An important role in the accumulation of therapeutic nucleic acids in tumors is played by the effect of enhanced permeability and retention (EPR), which is caused by higher vascular permeability of the tumor and deterioration of lymphatic drainage [16]. The data obtained showed that an increase in the molecular weight of the conjugate does not prevent its exit from the vessels and accumulation in the ornaments, and in the case of a tumor, provides a more effective retention in the target organ. In order to assess how the increase in molecular weight affects the circulation time of the drug in the bloodstream, we carried out a determination of the concentration dynamics of Ch-siRNA and Ch-TsiRNA-2 in plasma. In the first 15 min, a rapid decrease in the concentration of drugs occurred, and this decrease in concentration was especially pronounced for Ch-TsiRNA. The subsequent observations showed similar dynamics of elimination for both preparations at all time points up to practically undetectable values after 24 h. The concentration of Ch-TsiRNA-2 at all time points was significantly lower than the concentration of Ch-siRNA (Figure 2C). This difference in elimination rate may be due to the fact that although TsiRNA has a higher molecular weight than siRNA, it is still below the filtration limit in the kidneys and does not give an advantage to the conjugate. On the other hand, less hydrophobicity and longer length of RNA per cholesterol molecule can reduce the efficiency of complex formation with lipoproteins and other blood proteins [3,17], which are important for increasing the circulation time of cholesterol conjugates of siRNA in the bloodstream. However, our data showed, that the accumulation of cholesterol-modified siRNA in the tumor depends more on the EPR effect, whose effectiveness is influenced by the molecular weight of the delivered drugs. Thus, in the case of trimeric siRNA, a three times greater molecular weight allows it to accumulate in the tumor more effectively (Figure 2B) despite its relatively short time of presence in the blood (Figure 2C).

The silencing activity of Ch-siRNA and Ch-TsiRNA-1 was determined by suppressing the expression of the *MDR1* gene in tumor xenograft KB-8-5 in SCID mice by determining the level of the target gene product P-glycoprotein using Western blot analysis. According to silencing activity data, despite a similar biodistribution pattern and more efficient accumulation in organs and tumors, Ch-TsiRNA-2 did not silence the *MDR1* gene 24 h after i.v. administration of 8.5 µg/g (Figure 2D), and silencing activity remained at control levels. However, the same concentration of Ch-siRNA reduced the level of P-glycoprotein (*MDR1* gene product) by 60%.

The lack of correlation between the accumulation in the organ and the silencing activity of cholesterol conjugates of siRNA of different lengths can be attributed to the fact that, despite the effective retention in the organ, the trimeric Ch-TsiRNA lags mainly in the intercellular space and does not penetrate sufficiently into the cytoplasm of the cell. Increased accumulation in the organs and in the tumor, by itself, shows that an increase in the molecular weight can be an effective approach to control biodistribution and delivery to the target organ. Additional efforts are required to increase the bioavailability of such conjugates and to ensure their penetration into the cells and their subsequent exit from the endosomes into the cytoplasm.

Conjugation of siRNAs with molecules for which natural transport mechanisms exist, such as lipophilic molecules [3], antibodies [17,18,19], aptamers [20], N-acetylgalactose [4], peptides [21], or other ligands is a promising approach to improve their bioavailability. Furthermore, an increase in the molecular weight of siRNA conjugates is considered as one of the approaches to lengthening the time of their circulation in the bloodstream and, accordingly, increase the accumulation in target organs [22]. An increase in the molecular weight of the conjugate can be achieved both by increasing the size or number of copies of the transport ligand [23], and the use of longer interfering RNAs [24]. The latter approach was successfully implemented in [24], where chemical conjugation was used for siRNA multimerization and it was shown that these multimers can be delivered in vivo by a GalNAc ligand. The specificity of our strategy is that the modification of both parts of the conjugate—lengthening siRNAs and using a ligand capable of forming complexes with blood components—is aimed at improving its pharmacodynamics. The obvious limitation of our strategy is related both to the need to ensure a balance between a decrease in the efficiency of penetration into cells and an increase in the efficiency of accumulation in the target organ. Since the properties of vasculature are one of the most important factors determining the efficiency of accumulation in a particular organ, optimization of the conjugate size is required for each target organ, while it is not obvious that this approach will have an advantage for the number of organs. In the case of the conjugates described in this article, the enhanced accumulation of conjugates is apparently provided by the effect of enhanced permeability and retention in the tumor [16], which opens up the possibility of concentrating efforts on their design for oncology purposes.

One of the important requirements for the development of P-glycoprotein inhibitors is the specificity of their action, since the suppression of the synthesis of this gene in untargeted tissues can lead to increased toxicity of chemotherapy and strong side effects of antitumor treatment. For example, reduced P-glycoprotein activity in the blood-brain barrier could lead to abnormally increased accumulation of drugs in the brain and undesired side effects [25]. Since that, it is very important that accumulation of Ch-siRNA in the brain, one of the most important organs, is non-detectable (data not shown). Thus, Ch-siRNA is a promising agent for creating on its basis agents for suppressing the expression of P-glycoprotein in clinical practice.

## 3. Materials and Methods

### 3.1. Synthesis of Monomeric and Trimeric siRNAs, Their Cholesterol-Containing Analogues and Duplex Annealing

The 2’-OMe-modified sense and antisense strands of siRNAs and TsiRNAs were synthesized on an automatic ASM-800 DNA/RNA synthesizer (Biosset, Novosibirsk, Russia) using ribo- and 2’-O-methylribo β-cyanoethyl phosphoramidites (Glen Research, Brook Park, OH, USA). A combination of H-phosphonate and phosphoramidite methods was applied to synthesize 5’-cholesterol conjugates of the sense strands via C6 linker as previously described [10]. Oligoribonucleotides and their conjugates were purified by denaturing polyacrylamide gel electrophoresis (PAGE) and isolated as sodium salts. The purified oligoribonucleotides were characterized by MALDI-TOF mass spectrometry on a PEFLEX III spectrometer (Bruker Daltonics, Bremen, Germany). The monomeric and trimeric anti-*MDR1* siRNA sequences are listed in Table 1. Control siRNA (siSCR) has no significant homology to any known mouse, rat, or human mRNA sequence (Table 1). siRNA duplexes were obtained via annealing of the antisense and sense strands at equimolar concentrations in buffer A (15 mM HEPES-KOH pH 7.4, 50 mM potassium acetate, and 1 mM magnesium acetate), which were stored at −20 °C.

### 3.2. Cell Culture

A multiple drug-resistant human cell line KB-8-5 growing in the presence of 300 nM vinblastine was generously provided by Prof. M. Gottesman (NIH, USA). KB-8-5-MDR-GFP and KB-3-1-MDR-GFP cell lines expressing the fragment of the *MDR1* mRNA and short-lived turboGFP mRNA in a single transcript, were obtained by lentiviral transduction as previously described [13]. The cells were grown in Dulbecco’s modified Eagle’s medium (DMEM), supplemented with 10% fetal bovine serum (FBS), 100 U/mL penicillin, 100 μg/mL streptomycin and 0.25 μg/mL amphotericin at 37 °C in a humidified atmosphere containing 5% CO_2_/95% air.

### 3.3. Gene Silencing Assay

One day before the experiment, KB-8-5-MDR-GFP or KB-3-1-MDR-GFP cells in the exponential phase of growth were plated in 48-well plates at a density of 2.5 × 10^4^ cells/well. After 24 h, the growth medium was replaced by fresh serum-free DMEM (200 µl/well). siRNAs were added to the cells in 50 µL of Opti-Mem to give the final concentration varying from 1 to 5 µM. Alternatively, the cells were transfected with siRNAs (0.1–100 nM) using Lipofectamine 2000 (Invitrogen, Waltham, MA, USA) according to the manufacturer’s protocol (1 µL per well). Three days post-transfection, the cells were trypsinized and 8000 cells from each sample were analyzed using the NovoCyte flow cytometer (ACEA Biosciences, San Diego, CA, USA). Silencing activity data were obtained using mean fluorescence intensity values of cells measured in relative fluorescent units (RFU) and equation *MDR1*-*GFP* (%) = (RFU_sample_ (KB-8-5-MDR1-GFP) − RFU (KB-8-5))/(RFU_control_ (KB-8-5-MDR1-GFP) − RFU (KB-8-5)) × 100% and same with KB-3-1-MDR1-GFP cells, untreated cells were used as a control.

### 3.4. Stem-loop RT-PCR Cellular Accumulation Assay

KB-8-5 cells preparation and addition of siRNAs were similar to the protocol followed in the gene silencing assay. Four hours after siRNA addition, the cells were lysed using Triton X-100 (PanReac AppliChem, Barcelona, Spain) as previously described [26]. siRNA-specific stem-loop RT-PCR assays were designed according to the instructions of Czimmerer et al. [27] using UPL-probe based stem-loop quantitative PCR assay design software (freely available online at http://genomics.dote.hu:8080/mirnadesigntool). The sequences of the primers targeting siRNA were as follows: stem-loop primer, 5′- GTTGGCTCTGGTGCAGGGTCCGAGGTATTCGCACCAGAGCCAACCATCAG-3′, PCR forward primer, 5′- GTTGGGGATATACAACTTGTCA -3′, PCR reverse primer, 5′- GTGCAGGGTCCGAGGT -3′. The sequences of the primers for TsiRNA-2 were as follows: stem-loop primer, 5′-GTTGGCTCTGGTGCAGGGTCCGAGGTATTCGCACCAGAGCCAACAGCAGA-3′, PCR forward primer, 5′- ATATACAACTTGTCAAGCCAAATCC-3′, PCR reverse primer, 5′- AGAGGCCGCTGTTCGTTT -3′. Synthesis of cDNA and stem-loop PCR was carried out using SuperScript III Reverse Transcriptase (Thermo Fisher Scientific, Waltham, MA, USA); and qPCR mix was assembled using BioMaster qPCR SYBR Blue (Biolabmix, Novosibirsk, Russia). Serial dilutions of siRNA and its conjugates were added to the portions of cellular lysates (from 1.5 × 10^4^ cells) to obtain calibration curves.

### 3.5. Mice

All animal procedures were carried out in strict accordance with the recommendations for proper use and care of laboratory animals (ECC Directive 86/609/EEC). The protocol was approved by the Committee on the Ethics of Animal Experiments of the Administration of the Siberian Branch of the Russian Academy of Sciences (22.11 from 30.05.2014). The experiments were conducted at the Center for Genetic Resources of Laboratory Animals at the Institute of Cytology and Genetics, Siberian Branch, Russian Academy of Sciences (RFMEFI61914X0005 and RFMEFI62114X0010). Eight- to 10-week-old female SCID (SHO-*Prkdc^scid^Hr^hr^*) mice with an average weight of 20–22 g from the Center for Genetic Resources of Laboratory Animals at the Institute Cytology and Genetics SB RAS were used. In addition, 8- to 10-week-old male C57Bl mice with an average weight of 20–22 g were obtained from the vivarium of the Institute of Chemical Biology and Fundamental Medicine SB RAS. Mice were housed in groups of 8–10 individuals in plastic cages with free access to food and water; daylight conditions were normal.

### 3.6. Stem-Loop RT-PCR Analysis of siRNA and TsiRNA Concentration in Mice Blood and Internal Organs After Intravenous Administration

C57Bl mice were i.v. injected with 2.5 nmole of cholesterol-containing conjugates of siRNA or TsiRNA, and blood samples were collected 5 min, 1, 2, 4, and 24 h after the injection. Mice were sacrificed 24 h after injection and siRNA was extracted from organs using Triton X-100 according to [26]. The blood was centrifuged, plasma samples were diluted by 10 V of 0.25% Triton X-100 and heated to 95 °C for 10 min, then cooled on ice and centrifuged for 10 min (4 °C; 12,000 rpm). In addition, the mice were sacrificed 24 h after injection, and siRNA and TsiRNA were extracted from organs using Triton X-100. The collected supernatants were heated to 95 °C and immediately added to the RT mixture in a ratio of 2 μL of the supernatant per 38 μL of the Master mix. Serial dilutions of siRNA and its conjugates were added to the same sample volumes of mice blood or tissue homogenate as processed similarly for the construction of calibration curves. siRNA-specific stem-loop RT-qPCR assays were carried out as described above (Section 3.4).

### 3.7. Analysis of siRNA and TsiRNA Accumulation and Silencing Activity in KB-8-5 Xenograft Tumors in SCID Mice After Intravenous Administration

Tumors were initiated in mice by inoculating 10^6^ KB-8-5 cells in 200 mL of 0.9% saline solution subcutaneously into the right side of the mice and were allowed to grow to approximately 1 cm^3^ volume. Five mice per group were i.v. injected with 2.5 nmole siRNA or TsiRNA or its cholesterol-containing conjugates, and two mice from each group were sacrificed after 24 h. The tumors were excised and cut into 100–200 mg sections, from which siRNA or TsiRNA was extracted and quantified as described in Section 3.6. Three mice per group were sacrificed six days after injection and the level of P-glycoprotein in KB-8-5 tumors was evaluated by Western blotting. The sections were weighed and homogenized using 300 µL of RIPA buffer per 100 mg of tumor tissue. The samples were stirred for 30 min at 4 °C, and then they were cleared by centrifugation at 10,000× *g* for 10 min (4 °C). Supernatants were diluted by two volumes of sample buffer (Sigma-Aldrich, St. Louis, MO, USA), and 10 μL of each sample was loaded onto a 10% SDS/polyacrylamide gel and then separated at 60 mA for 1 h. The proteins were transferred from PAAG to PVDF membranes (Millipore, San Diego, CA, USA) using SemiPhor (Hoefer, Holliston, MA, USA), then the membrane was blocked for 1 h in 1% non-fat dried milk in PBS. The membranes were incubated overnight with monoclonal anti-P-glycoprotein and anti-β-actin antibodies (Sigma-Aldrich, St. Louis, MO, USA) at 1:800 and 1:7000 dilutions, respectively. After the membranes were washed in PBS with 0.1% Tween-20, they were subsequently incubated for 1 h with secondary rabbit anti-mouse antibodies conjugated with peroxidase (Abcam, Eugene, Oregon). Visualization was performed using a Western Blotting Chemiluminescent Reagent Kit (Abcam, OR, USA). Human β-actin protein was used as an internal control. Data were analyzed using GelPro 4.0. software (Media Cybernetics, Rockville, Maryland).

### 3.8. Statistical Analyses

The variables were expressed as the mean ± standard deviation (SD). Mean values were considered significantly different when *p* < 0.05, using the Student’s *t*-test (*n* = 3).

## Figures and Tables

**Table 1:** Sequences of siRNAs and calculated IC_50_ values for *MDR1-GFP* gene silencing after transfection into KB-8-5-MDR1-GFP cells by Lipofectamine 2000.

Designation	Sequence ^1^	IC_50_, nM
siRNA	5′-GGCU**U**GA**C**AAGU**U**G**U**A**U**A**U**GG-3′ 3′-AA**C**CGAA**C**UG**U**UCAA**C**A**U**A**U**A-5′	3.8 ± 3.5
Ch-siRNA	Ch-5′-GGCU**U**GA**C**AAGU**U**G**U**A**U**A**U**GG-3′ 3′-AA**C**CGAA**C**UG**U**UCAA**C**A**U**A**U**A-5′	29 ± 3
Ch-siSCRm	Ch-5′-**C**AAGUCUCG**U**A**U**G**U**AG**U**GGUU-3′ 3′-UUG**U**UCAGAGCA**U**A**C**A**U**CA**C**C-5′	
TsiRNA-1	5′-GGCU**U**GA**C**AAGU**U**G**U**A**U**A**U**GGGGCU**U**GA**C**AAGU**U**G**U**A**U**A**U**GGGGCU**U**GA**C**AAGU**U**G**U**A**U**A**U**GG-3′ 3′-AA**C**CGAA**C**UG**U**UCAA**C**A**U**A**U**AAACCGAA**C**UG**U**UCAA**C**A**U**A**U**AAACCGAA**C**UG**U**UCAA**C**A**U**A**U**A-5′	0.65 ± 0.14
Ch-TsiRNA-1	Ch-5′-GGCU**U**GA**C**AAGU**U**G**U**A**U**A**U**GGGGCU**U**GA**C**AAGU**U**G**U**A**U**A**U**GGGGCU**U**GA**C**AAGU**U**GUA**U**A**U**GG-3′ 3′-AA**C**CGAA**C**UG**U**UCAA**C**A**U**AUAAA**C**CGAA**C**UG**U**UCAA**C**A**U**A**U**AAA**C**CGAA**C**UG**U**UCAA**C**A**U**A**U**A-5′	16 ± 10
TsiRNA-2	5′-**C**AGAGGCCGC**U**GUUCGUU**U**GAGCGCGAGGUCGGGA**U**GGAUUUGGCU**U**GA**C**AAGU**U**GUA**U**A**U**GG-3′ 3′-UCG**U**CUCCGGCGA**C**AAGCAAA**C**UCGCGCUCCAGCCCUA**C**CUAAACCGAA**C**UG**U**UCAA**C**A**U**A**U**A	13 ± 7
Ch-TsiRNA-2	Ch-5′-**C**AGAGGCCGC**U**GUUCGUU**U**GAGCGCGAGGUCGGGA**U**GGAUUUGGCU**U**GA**C**AAGU**U**G**U**A**U**A**U**GG-3′ 3′-UCG**U**CUCCGGCGA**C**AAGCAAA**C**UCGCGCUCCAGCCCUA**C**CUAAACCGAA**C**UG**U**UCAA**C**A**U**A**U**A–5′	87 ± 13

^1^ Ch—cholesterol conjugated via aminohexyl linker; 2′-O-methyl-modified nucleotides are highlighted in bold and underlined.

**Table 2:** Strands of small interfering RNAs (siRNA) conjugates.

Designation ^1^	Strand ^1^	Mass Calculated	Mass Found
siRNA	Sense	6838.2	6838.1
	Antisense	6732.2	6732.4
Ch-siRNA	Ch-Sense	7428.8	7428.7
	Antisense	6732.2	6732.4
Ch-siSCRm	Ch-Sense	7351.6	7352.2
	Antisense	6687.1	6688
TsiRNA-1	Sense	20,682.5	20,684.5
	Antisense	20,320.6	20321
Ch-TsiRNA-1	Ch-Sense	21,229.7	21,230.7
	Antisense	20,320.6	20321
TsiRNA-2	Sense	20,547.1	20,545.1
	Antisense	20135	20,134.3
Ch-TsiRNA-2	Ch-Sense	21,138.7	21137
	Antisense	20,135	20,134.3

^1^ Ch—cholesterol conjugated via aminohexyl linker to 5′ end.
